# A single screen-printed electrode in tandem with chemometric tools for the forensic differentiation of Brazilian beers

**DOI:** 10.1038/s41598-022-09632-9

**Published:** 2022-04-04

**Authors:** Yhan S. Mutz, Denes do Rosario, Luiz R. G. Silva, Diego Galvan, Bruno C. Janegitz, Rafael de Q. Ferreira, Carlos A. Conte-Junior

**Affiliations:** 1 Graduate Program in Food Science (PPGCAL), Institute of Chemistry (IQ), Federal University of Rio de Janeiro (UFRJ), Cidade Universitária, Avenida Athos da Silveira Ramos, n. 149, Bloco A, 5° andar, Rio de Janeiro, RJ 21941-909 Brazil; 2 Center for Food Analysis (NAL), Technological Development Support Laboratory (LADETEC), Federal University of Rio de Janeiro (UFRJ), Cidade Universitária, Rio de Janeiro, RJ 21941-598 Brazil; 3 Chemistry Department, Federal University of Espírito Santo, Vitoria, ES 29075-910 Brazil; 4 Department of Nature Sciences, Mathematics and Education, Federal University of São Carlos, Araras, SP 13600-970 Brazil; 5 Postgraduate Program in Material Science (PPGCM-So), Federal University of São Carlos, Sorocaba, SP 18052-780 Brazil

**Keywords:** Electrochemistry, Biochemistry

## Abstract

In the present study a single screen-printed carbon electrode (SPCE) and chemometric techniques were utilized for forensic differentiation of Brazilian American lager beers. To differentiate Brazilian beers at the manufacturer and brand level, the classification techniques: soft independent modeling of class analogy (SIMCA), partial least squares regression discriminant analysis (PLS-DA), and support vector machines discriminant analysis (SVM-DA) were tested. PLS-DA model presented an inconclusive assignment ratio of 20%. On the other hand, SIMCA models had a 0 inconclusive rate but an sensitivity close to 85%. While the non-linear technique (SVM-DA) showed an accuracy of 98%, with 95% sensitivity and 98% specificity. The SPCE-SVM-DA technique was then used to distinguish at brand level two highly frauded beers. The SPCE coupled with SVM-DA performed with an accuracy of 97% for the classification of both brands. Therefore, the proposed electrochemicalsensor configuration has been deemed an appropriate tool for discrimination of American lager beers according to their producer and brands.

## Introduction

Beer is one of the most popular alcoholic beverages worldwide, with a US$ 612 billion revenue in 2021 and a 10% annual growth projected for this market between 2021 and 2025^1^. In 2018, Brazil was the third-biggest producer, with 14 billion liters, only behind China and United States^2^. Similar to the global market, the Brazilian beer market is dominated by a few large breweries. Currently, three major manufacturers account for 96% of the Brazilian beer market share with several brands each, where the most commercialized style is the American lager^3^. In such a competitive market, beers from these manufacturers are sold at higher values due to their effective market presence, consumer loyalty, and the quality attributed to the brand's name. However, these characteristics make these brands subject to fraud practices.

Due to reduced surveillance during the pandemic, recent reports revealed an increasing trend in beer fraud and counterfeiting practices^4^. A frequently spotted type of fraud is switching the label and cap of lower-value brands for higher-value ones, which is easy and effective as a counterfeit practice^5–7^. Detection of this type of fraud is analytically challenging as the distinctions among beers from the same style can be very subtle. Therefore, developing a fast, reliable, and portable technique to classify American lager beers from distinct manufacturers and brands is necessary to ensure product security. However, the challenge of classification amongst the same style required for detecting fraud has only been approached with advanced analytical techniques such as ^1^H NMR^8^ and paper spray mass spectrometry^9^. Although these techniques are very sensible, they rely on expensive equipment, organic solvents, and lack portability. In this regard, electrochemical sensors (ES's) are fast, reliable, and portable options for obtaining the fingerprint of several food matrices such as honey, beers, wines^10^. ES's are analytical systems composed of unspecific electrochemical sensors coupled with chemometric techniques to identify the characteristics of a complex system^10^.

The use of ES in beer science is described for classification among beers from distinct styles^11–13^ and distinct raw materials^14^. Since each beer will present chemical variation related to its manufacturing process and employ raw materials, the use of ES's emerges as a viable option for forensic tasks^15^. Indeed, in our previous work, we developed an electronic tongue based on four distinct commercially available screen-printed electrodes (SPE's) and chemometric techniques capable of discriminating between Premium and standard American lager beers^14^. However, from the analytical point-of-view, discrimination between manufacturer and brand level is a higher challenge, once discrimination between beer types (as Premium and Standard American lagers) may rely on the distinction between the raw materials. Moreover, to discriminate between beers of the same style from distinct brands offers great forensic potential. Therefore, regarding the need for rapid, reliable, and portable methods with forensic potential for beer classification, the present study evaluates using a voltammetric electrochemical sensor coupled with classification methods to discriminate American lagers from the three major Brazilian producers.

## Material and methods

### Beer samples

In total, 253 beer samples were purchased at local supermarkets in the metropolitan region of Espírito Santo State (Brazil). The dataset comprised 18 different brands from the three major commercialized groups in the Brazilian market. In detail, 99 samples from six brands of Manufacturer A, 61 samples comprising five brands from manufacturer B, 58 samples from four brands from manufacturer C. Moreover, 35 samples belonging to three other brands from a smaller manufacturer were included as manufacturer D to test the model capacity to rule out possible counterfeit candidates. The number of samples discriminated by each brand and manufacturer is given in Table S1.

### Instrumentation and voltammetric measurements

Cyclic voltammetry measurements were performed on a portable potentiostat/galvanostat μSTAT 400 (Metrohm DropSens, Oviedo, Spain) controlled by Dropview 8400® software using a disposable SPCE made by Metrohm-Dropsens (Oviedo, Spain). The SPCE used were a DS-110 carbon working electrode with dimensions of 3.4 × 1 × 0.05 cm containing three electrodes printed on the same planar ceramic platform: a working electrode, a pseudo reference electrode (Ag/AgCl), and an auxiliary electrode manufactured in the same material as the working electrode. In this work, the use of supporting electrolytes was not necessary once the samples (beers) presented electrolytes that make electrochemical measurements possible^14^.

Before electrochemical measurements, the beer samples were opened for 30 min for the removal of excess CO_2_. Voltammetric measurements were performed at room temperature (25 ºC) in triplicate, dropping a 40 μL aliquot on the surface of the electrode. The cyclic voltammetry scan was performed following the settings from a previous experiment^14^, using the range of between − 1.0 V and 1.0 V, with a scan rate of 100 mV s^−1^, totaling 40 s per scan, without the need for support electrolyte.

### Chemometric procedures

#### Data treatment

The beer samples voltammograms were concatenated in a matrix (253 × 2000), with each line corresponding to a sample and the columns corresponding to the current signals. The dataset was then preprocessed only by mean-centering^14^. To build the classification models according to the manufacturer, the samples were labeled for each of the four manufacturing groups (Manufacturer A, Manufacturer B, Manufacturer C, Manufacturer D). Furthermore, the samples were labeled as either Brand X, Brand Y, or other for the brand challenge. All statistical analyses were performed using the Matlab 2020a software® (The MathWorks Inc., Natick, USA).

#### Classification techniques

The linear classification techniques used were partial least squares discriminant analysis (PLS-DA) and soft independent modeling of class analogies (SIMCA) using the classification toolbox of the Milano chemometrics and QSAR research group^16^. The non-linear technique support vector machines discriminant analysis (SVM-DA) was employed using the Gamma toolbox^17^.

SIMCA is a class modeling technique that relies on PCA to model a class with reduced dimensionality. SIMCA constructs individual models for each of the sample's categories. Therefore, the classes are independently modeled, new samples are predicted as belonging to the class or not. Further, class assignment relies on a distance measure to interpret whether the samples belong to the modeled class. In the implemented SIMCA, the distance measure combines the normalized T^2^ statistics and normalized Q residuals^16^.

A multi-class PLS-DA model was built to define class boundaries between the distinct categories simultaneously. The techniques focus on the dissimilarities between the different classes to find class belonging traits. PLS-DA uses the PLS2 algorithm to search for latent variables that maximize the correlation between independent and dependent variables. A class threshold is defined for each class to minimize the number of incorrect assignments. Thus, the samples are assigned to a class based on the probability of class belonging based on their estimated class value and calculated class threshold. Non-assignments in the present study are achieved if a sample estimate is higher than more than one class or lower than all of the class thresholds. In this case, the sample would be assigned to multiple classes or not at all. In any case, these two scenarios lead to an unassigned sample^16^.

SVM-DA is a non-linear discriminant technique. The technique employs kernels to map data from linearly inseparable problems into high dimensional feature spaces and then perform classification^18^. In the present study, the radial basis function (RBF) was chosen as the kernel function. In this technique, the dimensionality of the feature space that performs the samples separation is determined by the RBF γ parameter. At the same time, the complexity of the model is set by the penalty parameter C. Together, the γ and C parameters control a trade-off between the model generalization ability and its complexity^17^.

#### Optimization of the model parameters

The optimization of the number of principal components for SIMCA and latent variables for PLS-DA was done with tenfold cross-validation in the training dataset. For this cross-validation, the training set was divided into ten subgroups, and one subgroup at a time was removed from the data set and used for external validation for the constructed model. The number of components or latent variables that minimize the classification error was chosen at the end of the process.

The SVM parameter C and the RBF kernel parameter γ were simultaneously optimized by particle swarm optimization (PSO) using tenfold cross-validation to maximize the classifier performance (RMSECV values). PSO setup used as previously described by^19^ lower and upper bounds limits between 1 × 10^–8^ to 1 × 10^–3^ for γ and 1 × 10^0^ to 1 × 10^5^ for C.

#### Validation, and performance of the models

To perform the external validation, the dataset (253 beer samples) was split into two subsets: a training set with 70% of the samples of each class (178 samples) and test set with the remaining 30% (75 samples).. The datasets were separated using the duplex algorithm^20,21^. Then, the validation was performed by calculating the performance parameters of the obtained classification models.

The performance for the constructed classification models evaluated were: sensitivity (Eq. 1), specificity (Eq. 2), accuracy (Eq. 3), and inconclusive ratio-IR (Eq. 4).

1 $$Sensitivity = \frac{TP}{{TP + FN}}$$

2 $$Specificity = \frac{TN}{{TN + FN}}$$

3 $$Accuracy = \frac{TN + TP}{{TN + TP + FN + FP}}$$

4 $$IR = \frac{NA}{N}$$

where: TP, FN, TN, and FP indicate the number of true positive, false negative, true negative, false-positive samples, respectively. Further, IR is the inconclusive rate, NA is the number of non-assigned samples, and N is the number of samples belonging to the class.

## Results and discussion

### Electrochemical profile of the beer manufacturers

**Figure 1:**
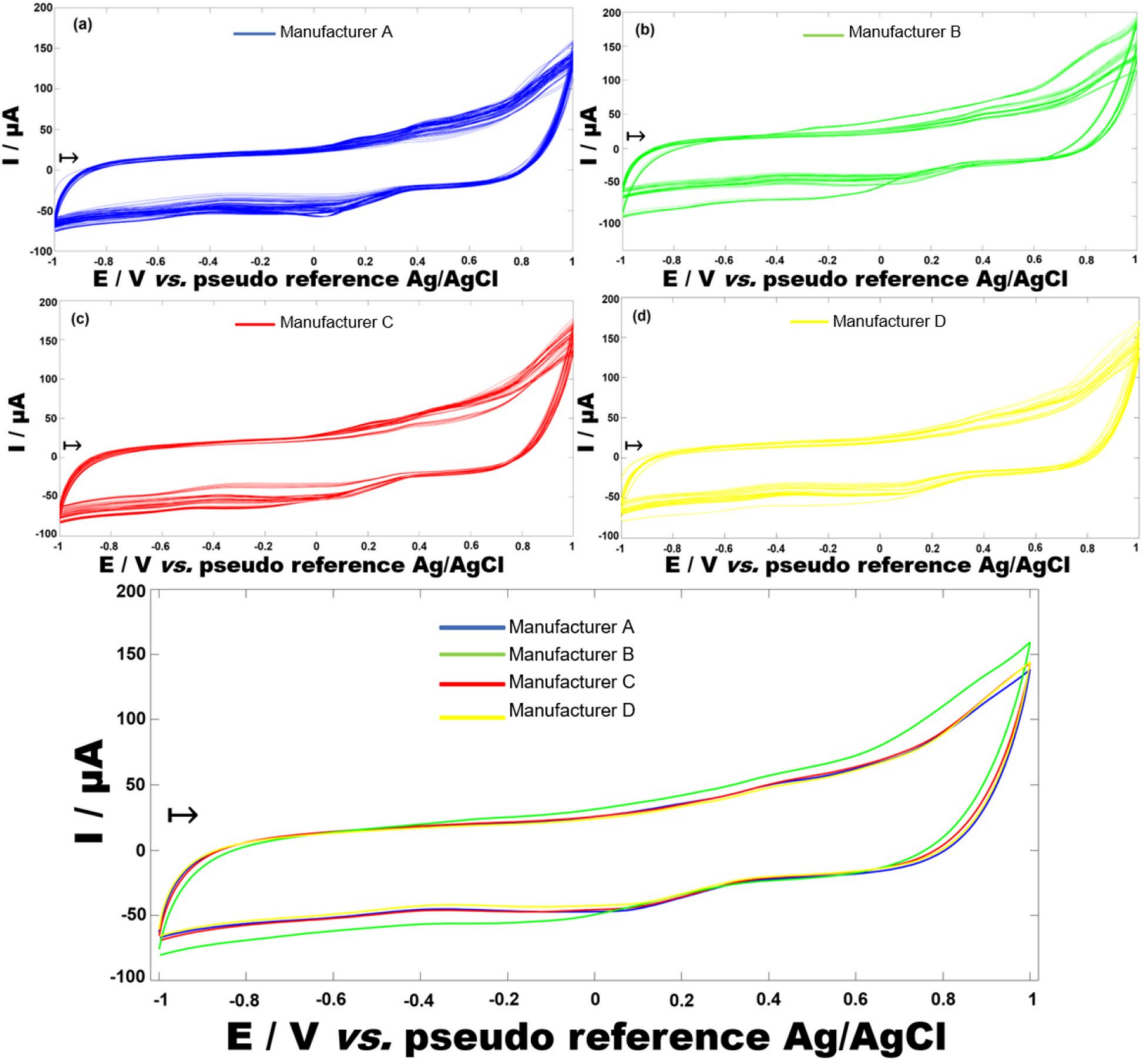
(**a**-**d**) All cyclic voltammograms of Brazilian American lager beer discriminated by manufacturers and (**e**) average cyclic voltammograms from the 253 beers obtained with screen-printed carbon electrodes. Scan rate: 100 mV s − 1. Scan direction ( →).

Cyclic voltammetry is the most used technique for acquiring qualitative information on the electrochemical properties of a food system. It consists of a linear scan of the potential of a working electrode, thus providing information on the redox process and electron-transfer reactions of a matrix^22^. As beer is a matrix that possesses several molecules susceptible to redox processes, the collected information becomes helpful for constructing classification models^14^. In the present study, the electrode used to obtain the electrochemical signals from the cyclic voltammetry was an SPCE. The adoption of an SPCE was based on results from our previous study, of beers discriminated according to their types^14^. Furthermore, following our previous study's findings, each disposable SPCE was used for 35 cyclic voltammograms, as it is their calculated lifetime.All voltammograms and average voltammograms of the samples discriminated by the manufacturer are shown in Fig. 1. Although a remarkable similarity between the voltammograms obtained can be observed (Fig. 1A–D), it is also noticeable that each manufacturer shows its peculiarities. The voltammograms of samples from Manufacturer A show a slight "peak" of reduction around 0.1 V in practically all samples, which does not appear with such intensity in the other groups of samples. The voltammograms from Manufacturer C showed a variability in current values from − 1 to − 0.2 V . Similarly, the voltammograms from Manufacturer B also presented current variation between − 1 V to − 0.2 V and 0.6 V to 1 V . These variations within the same manufacturers may be an indicator of a distinction between the brands from these manufacturers. Finally, the voltammograms obtained from "others" group samples present a suitable variety of profiles obtained, with samples generating responses similar to all three groups discussed above.

In Fig. 1E, it is observed that the average voltammograms from the manufacturers are similar with subtle distinctions, except for the Manufacturer B voltammogram that stands out with lower current values between − 1 V to 0 V and higher current values from 0.6 V to 1 V. Each beer is unique, varying on the type of fermentation, style, ingredients, and manufacturing process^15^.

In the present study, the challenge is to gather electrochemical information about beers of the same style from distinct producers. The electrochemical distinctions at the manufacturer or brand level between beers of the same style can be attributed to several electroactive molecules such as Na^+^/K^+^ adducts of oligosaccharides^9^, phenolic acids, esters, higher alcohols, and organic acids^8,9,23–25^. Therefore, as these classes of molecules can be detected by the employed cyclic voltammetry technique^14,26^, it can be assumed that difference in the voltammograms is related to their contents.

The content of organic acids and higher alcohols in beer relates to several distinctions in beer manufacturing. Higher alcohols are byproducts of yeast metabolism, either in anabolic or catabolic pathways^27^. As for the organic acids, although they may be a product of yeast metabolism, their majority is produced during the malt germination step^28^. Indeed organic acids are essential to beer flavor, as they directly influence its sourness^29^. Due to their importance, there are classification studies based on the beer's organic acid profile^8,28^. Furthermore, the distinction in phenolic acids, esters, aldehydes, ketones, and other electroactive molecules is multifactorial, from the raw ingredients employed to the brewing process variables its quality control^30^.

### Classification according to manufacturer

The task of classifying the beers according to their distinct manufacturers was approached with supervised pattern recognition techniques (PLS-DA, SIMCA, SVM-DA). Table 1 shows the classification performance by the manufacturer and by brands for the three applied algorithms. The performance parameters obtained by the model against the data of the training dataset can be found in Table S2.

**Table 1:** Performance parameters calculated with the test dataset for the classification models built for manufacturer and brand distinction.

Technique	Manufacturer	Test			
		Sensitivity	Specificity	Accuracy	IR
PLS-DA	Manufacturer A	0.95	0.92	0.83	0.20
	Manufacturer B	1.00	0.96	0.83	–
	Manufacturer C	0.43	1.00	0.83	–
	Manufacturer D	0.90	0.90	0.83	–
SIMCA	Manufacturer A	0.82	0.89	0.86	0.00
	Manufacturer B	0.84	0.96	0.93	0.00
	Manufacturer C	0.76	0.93	0.89	0.00
	Manufacturer D	1.00	0.95	0.96	0.00
SVM-DA	Manufacturer A	0.93	0.97	1.00	0.00
	Manufacturer B	1.00	0.98	0.94	0.00
	Manufacturer C	0.88	0.96	0.98	0.00
	Manufacturer D	1.00	1.00	0.96	0.00
SVM-DA	Brand X	0.90	0.98	0.97	0.00
	Brand Y	0.87	0.97	0.96	0.00
	Other brands	0.97	0.89	0.96	0.00

The first approach was made using linear classification methods such as PLS-DA and SIMCA. In the present study, the PLS-DA constructed model with four classes (Manufacturer A, Manufacturer B, Manufacturer C, and Manufacturer D) presented a prediction accuracy of 83%. It is worth mentioning that the PLS-DA technique weights down noisy spectral information but still keep it in the model. Therefore, PLS-DA is remarked as prone to overfitting^17^. Therefore, to check for overfitting in the built model, random permutations were performed on the manufacturer labels to assess the performance of random models. The random label models' performance and predicted variance (Q2Y) were inferior to the authentic model (Table S3). In addition, the model built with random labels had, on average a, 63% of non-assigned samples; Therefore, the overfitting of the model was ruled out.

The PLS-DA performance parameters were above or equal to 90% for all classes except for the Manufacturer C beers, which presented a sensitivity of 43% (Table 1). PLS-DA is a discriminant technique that focuses on the distinction among the defined classes to define a separation threshold^31^. The low sensitivity of this class may indicate a high intra-class variation, i.e., a distinct electrochemical profile among samples from Manufacturer C. On the other hand, the Manufacturer C samples distinction in their voltammograms compared to the other manufacturers (Fig. 1) corroborate the 100% specificity achieved in this class.

Nonetheless, the use of PLS-DA to classify the beer manufacturers were not considered satisfactory once the built model could not assign 15 out of the 75 samples of the test samples, leading to an inconclusive rate (IR) of 20%. Corroborating with our findings^8^ approached the classification of Brazilian beers by its manufacturers using ^1^H NMR spectroscopy and PLS-DA and also found a non-assignment rate of 6.89%. In the present study, with the adopted algorithm (classification toolbox^16^), non-assignments by the PLS-DA model are achieved when a sample is perceived as belonging to multiple classes or none of the classes at all^16^. Therefore, it can be implied that under the experimental conditions tested, the PLS-DA model did not have sufficient discriminative power to separate the manufacturers based on their electrochemical profile.

On the other hand, the SIMCA models did not present non-assigned samples, leading to an inconclusive rate of 0. In addition, the models achieved an accuracy between 86 and 96%, being superior to the PLS-DA model. In contrast to PLS-DA, a discriminant method, SIMCA is a one-class-classifier (OCC) technique that focuses on the class similarities to individually model class boundaries^32^. SIMCA models presented suitable values for specificity, with an average value of 93%. Specificity represents the model's ability to reject samples from other classes from being classified as the observed class^16^. This metric is specifically essential when dealing with authentication or forensic tasks as their objective is to rule out out-of-pattern samples caused by fraudulent practices^33^.

For this reason, the use of class modeling techniques is regarded^34^. Indeed, SIMCA is widely adopted to check the authenticity of food and beverages^32^. However, except for the group of other manufacturers, the SIMCA models' sensitivity was low. Therefore, pointing to an inefficient recognition of the samples as true belonging to the tested class.

**Figure 2:**
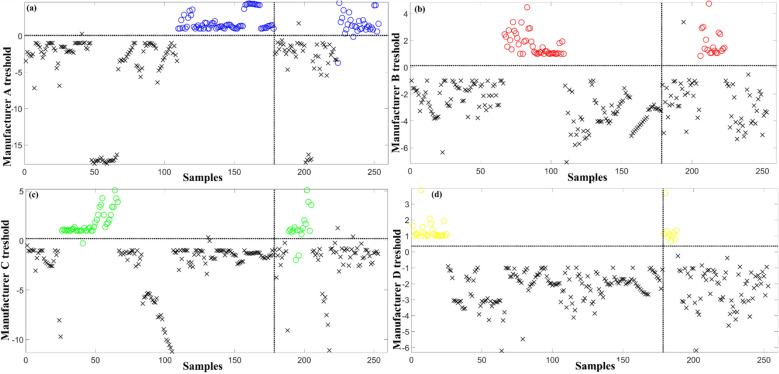
SVM-DA prediction showing the calculated threshold for discrimination (horizontal dashed line) of (**a**) Manufacturer A. (**b**) Manufacturer B. (**c**) Manufacturer C. (**d**) Manufacturer D. The vertical dashed line separates the training dataset (left side) and the test dataset (right side).

The overall low performance of the PLS-DA and SIMCA models can indicate that the brands could not be linearly separated. Therefore, SVM-DA, a non-linear classification technique, was employed. First, the SVM-DA parameters γ and C were adjusted using particle swarm optimization to minimize the value of RMSECV. The parameters were chosen after a tenfold cross-validation process to minimize classification error. The manufacturer model optimized parameter was γ of 8.90 × 10^–7^ and C of 4.85 × 10^3^, with an accuracy between 94 and 100% of the SVM-DA technique being superior to the linear methods (Table 1). SVM-DA separation also performed with an specificity superior than 96% and sensitivity from 88 to 100%. The model's prediction for the beer samples of all the defined classes can be seen in Fig. 2.

Our review showed that most studies that applied non-linear methods to the electrochemical sensing data had better classification or regression performance^10^. Although requiring higher computing power, these techniques allow for the separation of seemly inseparable overlapping classes^18^. Indeed, the use of SVM-DA for beer classification is successfully described in the literature as differentiation of geographical origin^24^ and authentication of Trappist beers^25^. Moreover, in accordance^12^, also found that SVM-DA led to lower (5.3%) classification error than PLS-DA (23.6%) when discriminating among beers with distinct fermentations using a carbon SPE and cyclic voltammetry.

SVM-DA maps the input data into higher dimensional feature spaces and then performs classifications^17,24^. In the present approach, the performed SVM-DA classification of multiple manufacturing brands was made with the one versus all (OVA) approach. In this approach, the targeted class is separated from the rest of the samples which are grouped as foreign samples. For being a discriminant technique, the use of the OVA approach means that the hyperplane designed to classify the beer brand considers all the other manufacturers as outsiders. Based on the model results, such a technique was considered suitable for the proposed classification task.

Such promising results raised the question of whether the developed SVM-DA-cyclic voltammetry ES is suitable to be employed in forensic tasks. The most simple and frequently related method of beer fraud in Brazil is to switch caps or labels of a lower-cost beer for a more expensive one^4,23^. Therefore, to test if the technique developed was indeed suitable for fraud detections, we tested its ability to differentiate beers at the brand level. To perform the brand challenge two frequently reported frauded brands within the Brazilian big producers, Brand X from Manufacturer A and Brand Y from Manufacturer C, were selected^5–7^.

The optimization of the SVM parameters for this challenge was performed for the manufacturer models. The optimum values obtained were γ of 3.52 × 10^–6^ and C of 1.48 × 10^1^. The tuning of the γ and C parameters defines the classification boundaries of the SVM model. While γ defines the influence of each selected support-vector, and therefore the smoothness of the classification surface, C controls the model complexity in a trade-off between the number of incorrect classifications and the model margin^17^. Thus, a higher C means that the dataset is reliable, and a higher penalty is given to classification error in exchange for a lower margin for the decision boundary is employed^35^. Therefore, for the beers classification, the brand SVM-DA model had a smoother classification boundary but a noisier dataset when compared to the manufacturer's model. Which can be attributed to the possibility of some of the sampled beers being electrochemically close to the profile of the Brand X and Brand Y studied beers.

**Figure 3:**
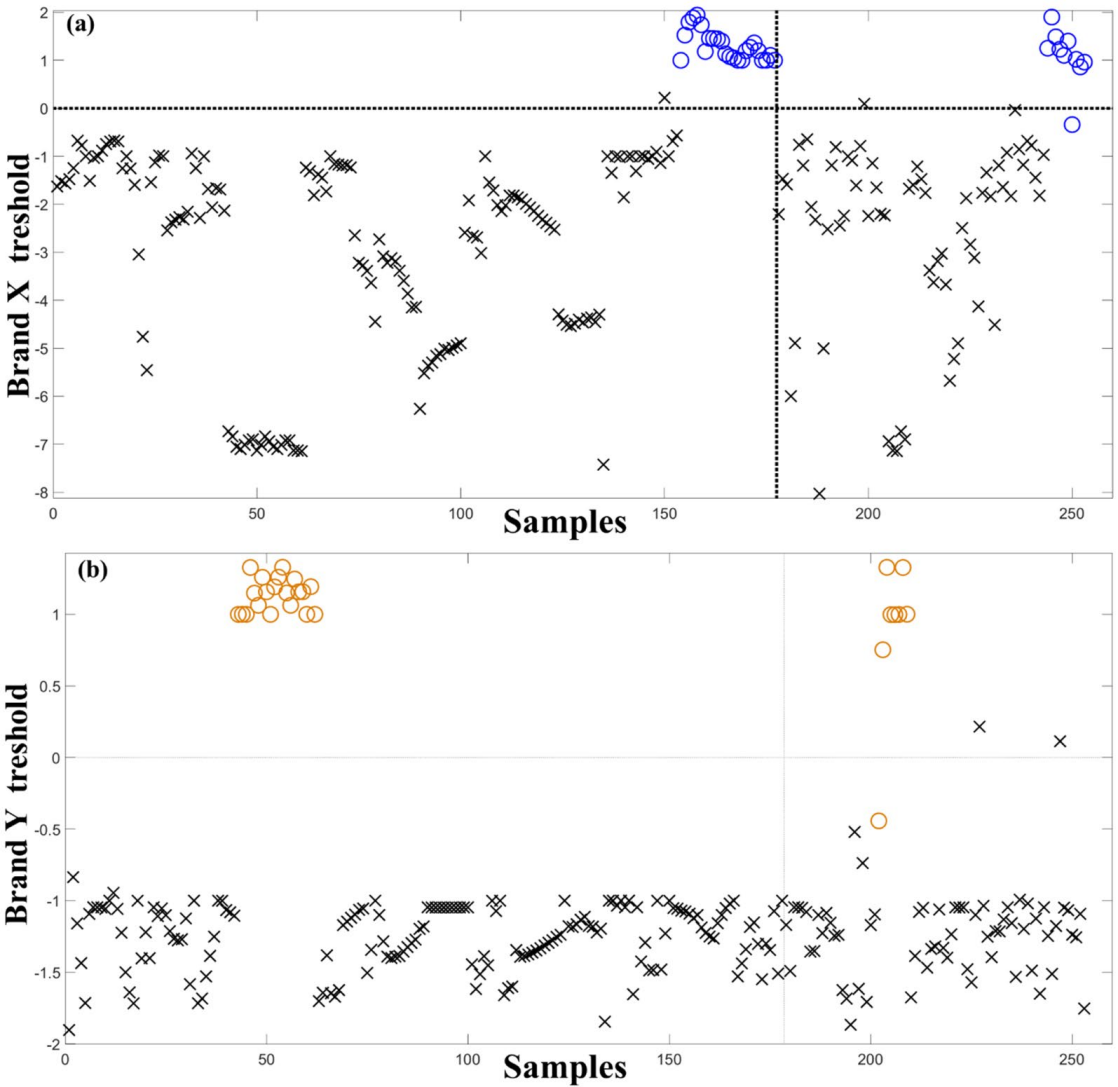
Samples plot showing the calculated threshold for discrimination (grey line) for the support vector machines discriminant analysis model. Blue circles are (**a**) Brand X beer samples, (**b**) Yellow circles are Brand Y beer samples, and black x's are other beer brands in the dataset; Samples above the grey line are classified as Brand X or Brand Y for the technique. Samples from the left side of the grey line are part of the training dataset and on the right side are part of the test set.

The SVM-DA model constructed for the brand challenge presented no un-assignments leading to an inconclusive rate of 0. The separation between the two tested brands versus other beers is shown in Fig. 3. Similar to the results found for the manufacturer test, the SVM-DA models presented high accuracy for the test dataset (97% for Brand X and 96% for Brand Y) (Table 1). Further, the sensitivity of the brand models is between 87 – 97%%, with specificity ranging from 89 to 97% (Table 1). Therefore, after being suitable for differentiation at the manufacturer level, a separation of the brand level was achievable with the SPCE-SVM-DA configuration developed for the ES and thus, expanding the application of the proposed sensor.

Several beer classification studies can be found in the scientific literature covering several characteristics of different manufacturers, raw materials, and origin designations. Table 2 presents a compilation of some studies that employ different analytical techniques in tandem with chemometric methods for beer classification. As it can be seen, the accuracy of the SPCE-SVM-DA models built in our study is among the highest values found in the literature for the classification with distinct or similar analytical techniques. Furthermore, it is noteworthy that the performance parameters for the constructed models in the present study were achieved with a dataset higher than the average studies found in the literature. The sampling of the dataset is of utmost importance, both in the representation of the total population and specific groups, as for validation of the constructed models, as a smaller number of samples may misrepresent or not be enough to validate a class behavior^21,36^. Therefore, although modeling a complex dataset, the finding for the present SPCE-SVM-DA protocol proved to be among the highest performances in the literature with validated findings for the models.

**Table 2:** Compilation of distinct analytical approaches combined with chemometrics for beer classification.

Instrument	Technique	Goal	Sample	Accuracy* (%)	Ref
Gas chromatography-mass spectrometry	HS-SPME-GC-TOFMS and ANN-MLP	Discriminate trappist class and specific brands from non-trappist	265 specialty beer samples	93.9–97	^37^
ISEs	Potentiometry and LDA	Discrimination of different commercial beer types	51 different brands and varieties of beer	81.9	^26^
Fluorescence and UV–Vis spectrophotometer	Spectroscopy and PCA-LDA data fusion	classification of canned samples of Chinese lager beers by manufacturer	135 canned beer samples from eleven Chinese manufacturers	78.5–86.7	^38^
Paper spray mass spectrometry	Paper spray mass spectrometry and OPS-PLS-DA	Differentiation of Brazilian American lager beers according to their brands	141 samples from four breweries	100	^9^
Spectrometer	^1^H NMR spectroscopy and PLSDA/SIMCA	Discriminate Standard and Premium Brazilian American lager beers	20 Premium American Lager and 20 Standard American Lager	91.6–100	^23^
Fluorescence spectrophotometer	EEM fluorescence and PARAFAC-kNN	Characterization and classification of Chinese beers from different manufacturers	108 canned beer samples from four major Chinese manufacturers	91.7	^39^
SPCE	Voltammetric and PLS-DA	Differentiation of Brazilian Premium american lager and Standard american lager	59 Premium american lagers and 54 Standard american lagers	94	^14^
SPCE	Voltammetric and SVM-DA	Differentiation of Brazillian Beer at manufacturer and brand level	253 beers from four major Brazilliam manufacturers	96–98	Present study

*Accuracy: Rate of correct classification in relation to an external test set; SPCE: screen-printed carbon electrode; ISE: Ion-selective-electrodes; SVM-DA: support vector chamiche discriminant analysis; LDA: linear discriminant analysis;EEM: excitation-emission matrix; NMR: Nuclear magnetic resonance; PARAFA: parallel factor analysis; kNN: k-Nearest neighbours: PCA: principal component analysis; PLSDA: partial least squares discriminant analysis; OPS: ordered predictors selection; HS-SPME: headspace solid phase micro extraction; ANN-MLP: artificial neural network with multilayer perceptrons.

Spectrometric techniques commonly result in the best pectrometric techniques commonly result in the best prediction results among the distinct chemometric approaches used to classify and discriminate beers for different purposes (Table 2). The efficiency of techniques relates to their high sensitivity, precision, and ability to identify a series of compounds that strongly help construct more accurate and robust chemometric models^40,41^. However, the use of such techniques and types of equipment has some disadvantages, such as high implementation cost, need for specialized handling and laboratories, time-consuming sample preparation, and low portability capacity. On the other hand, they are expensive techniques as compared with the electrochemical ones. Therefore, electrochemical techniques are presented as an alternative to the commonly used methods. They are fast, with a relatively low cost, easy to handle, and, as highlighted in the present study, with great potential for beer classification. Indeed, as depicted in Table 2, the two studies relating the use voltammetric ESs (our previous research and the present study) presented good classification accuracy. The evidence shows that the ES composed of SPCE coupled with PLS-DA was able to differentiate beers according to their styles^14^. The different malt used in the two types of beer led to the electrochemical distinctions captured by the ES^14^. Furthermore, with the with the further adoption of a non-linear algorithm (SVM-DA) the recognition of beer manufacturers and brands was achieved in the present study. The application of a non-linear method, which is more computationally demanding, is related to the finer distinction that exists when separating beer with the same styles from different manufacturers. Lastly, it should be noted that this technique is portable, with great potential for *in loco* analysis, could attract government agencies, consumers, and even breweries.

It should be mentioned that despite its advantages, commercial disposable electrodes have a limited useful life and cannot be used indefinitely. However, these disadvantages can be circumvented since these commercially available electrodes can be easily replaced by new ones as they have relatively low cost, thus granting reliable results disposable electrodes. Furthermore, novel alternatives include homemade electrodes (with paper, pet, and ink) to improve the accessibility and applicability of electrochemical sensors^42^.

## Conclusion

A novel approach based on a single screen-printed electrode coupled with SVM-DA for rapid, direct, and effective differentiation of Brazilian's American lager beers has been developed. The proposed configuration for an electrochemical sensor composed of SPCE with SVM-DA has been shown to predict with great specificity and sensitivity from the three major Brazilian manufacturers. Furthermore, the configured ES allowed for the differentiation at the brand level of two commonly frauded beers (Brand X and Brand Y). Therefore, the described portable ES capable of discriminating Brazilian American lagers at manufacturer and brand level may present a forensic potential against label and cap switch frauds.

## Supplementary Information

Supplementary Information.

## Data Availability

The datasets used and/or analyzed during the current study are available from the corresponding author on reasonable request.
