# Supplementary material for: A single screen-printed electrode in tandem with chemometric tools for the forensic differentiation of Brazilian beers
Source: Sci Rep. 2022 Apr 4;12:5630. doi: 10.1038/s41598-022-09632-9 (PMC8980006; doi:10.1038/s41598-022-09632-9)
Supplement: Supplementary file 1 — Supplementary Information. [file 41598_2022_9632_MOESM1_ESM.doc]

**Supplementary material**

**A single screen-printed electrode in tandem with the chemometric tool for the forensic differentiation of Brazilian beers**

Yhan S. Mutz^a,b^ , Denes do Rosario^a,b^, Luiz R.G. Silva^c,d,e^, Diego Galvan^b^, Bruno C. Janegitz^d,e^, Rafael de Q. Ferreira^c^, Carlos A. Conte-Junior^a,b^,*

^a^ Graduate Program in Food Science (PPGCAL), Institute of Chemistry (IQ), Federal University of Rio de Janeiro (UFRJ), Cidade Universitária, Rio de Janeiro, RJ, 21941-909, Brazil

^b^ Center for Food Analysis (NAL), Technological Development Support Laboratory (LADETEC), Federal University of Rio de Janeiro (UFRJ), Cidade Universitária, Rio de Janeiro, RJ, 21941-598, Brazil

^c^ Chemistry Department, Federal University of Espírito Santo, 29075-910, Vitoria, ES, Brazil

^d^ Department of Nature Sciences, Mathematics and Education, Federal University of São Carlos, 13600-970, Araras, São Paulo, Brazil

^e^ Postgraduate Program in Material Science (PPGCM-So), Federal University of São Carlos, 18052-780, Sorocaba, São Paulo, Brazil

^*^Corresponding author - conte@iq.ufrj.br (C. A. Conte-Junior), Instituto de Química, Avenida Athos da Silveira Ramos, n. 149, Bloco A, 5° andar, Rio de Janeiro, 21941-909, Phone +55-21-3938-7824

**
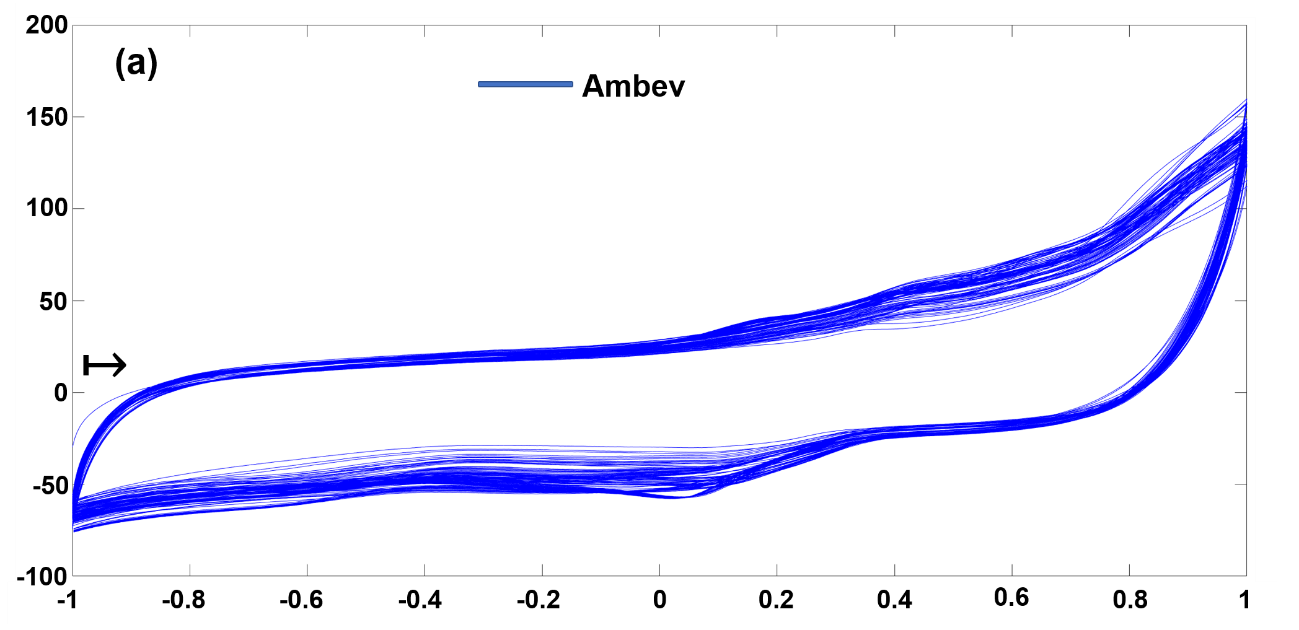
**

Fig. S1. Ciclyc voltamograms of different samples of beers from Ambev obtained with SPCE. Scan rate 100 mV s^−1^ and potential windows of −1 to 1 V.


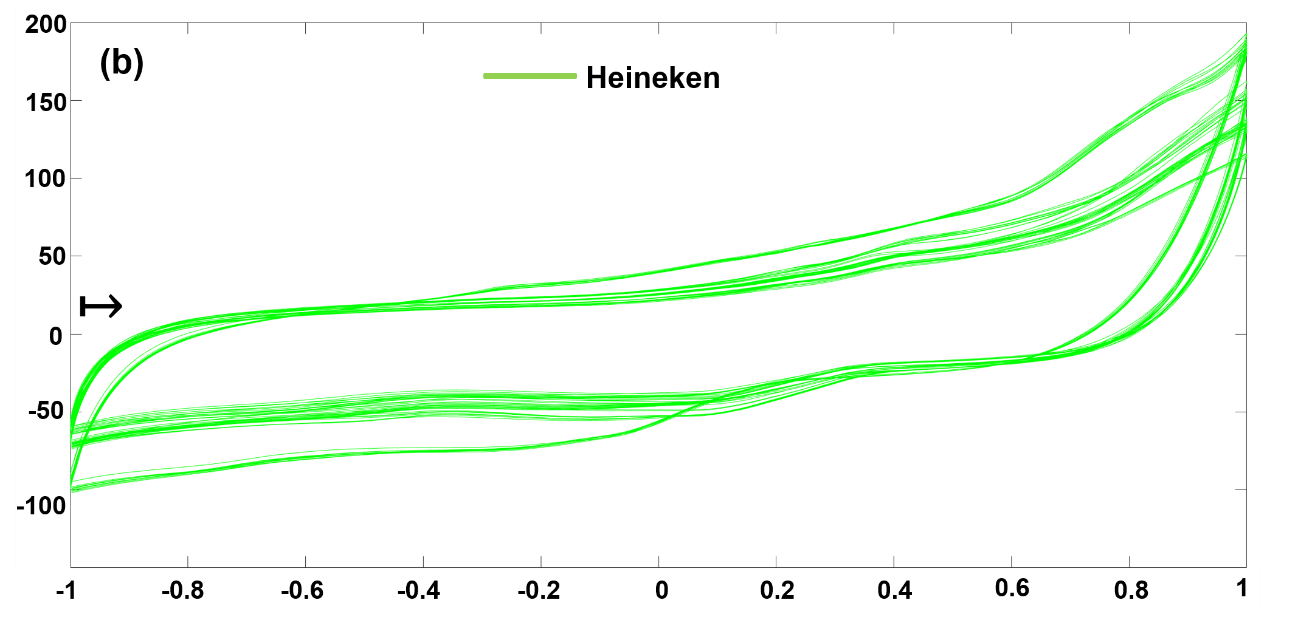


Fig. S2. Ciclyc voltamograms of different samples of beers from Heineken obtained with SPCE. Scan rate 100 mV s^−1^ and potential windows of −1 to 1 V.


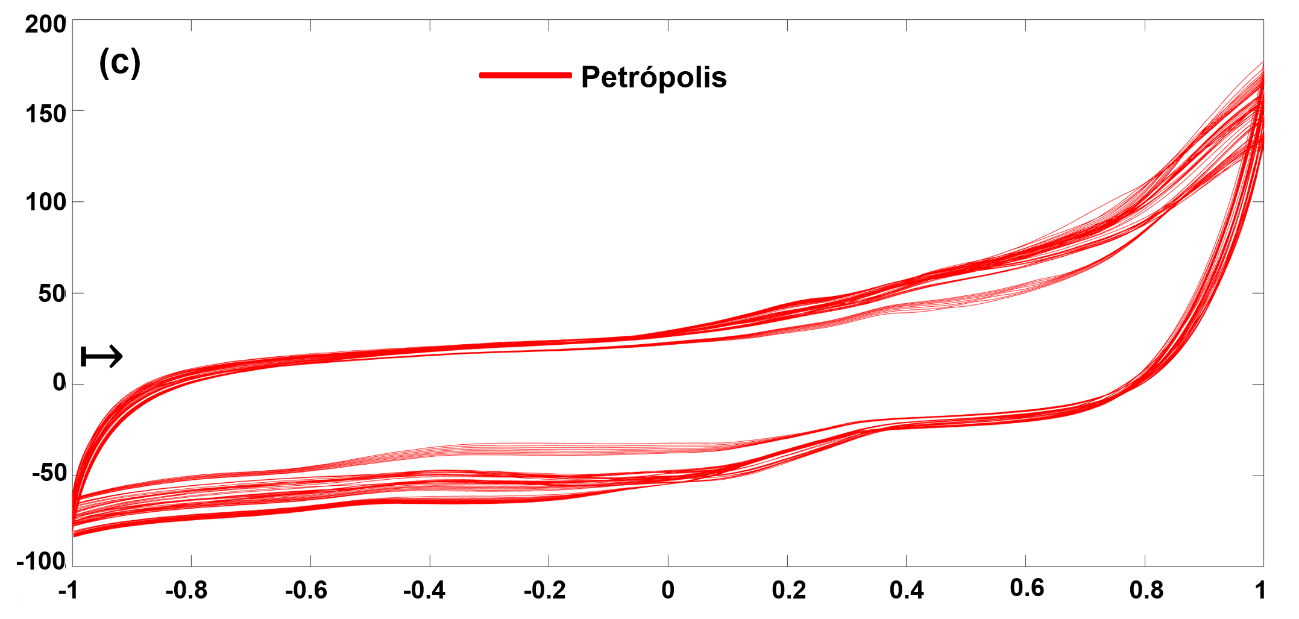


Fig. S3. Ciclyc voltamograms of different samples of beers from Petrópolis obtained with SPCE. Scan rate 100 mV s^−1^ and potential windows of −1 to 1 V.


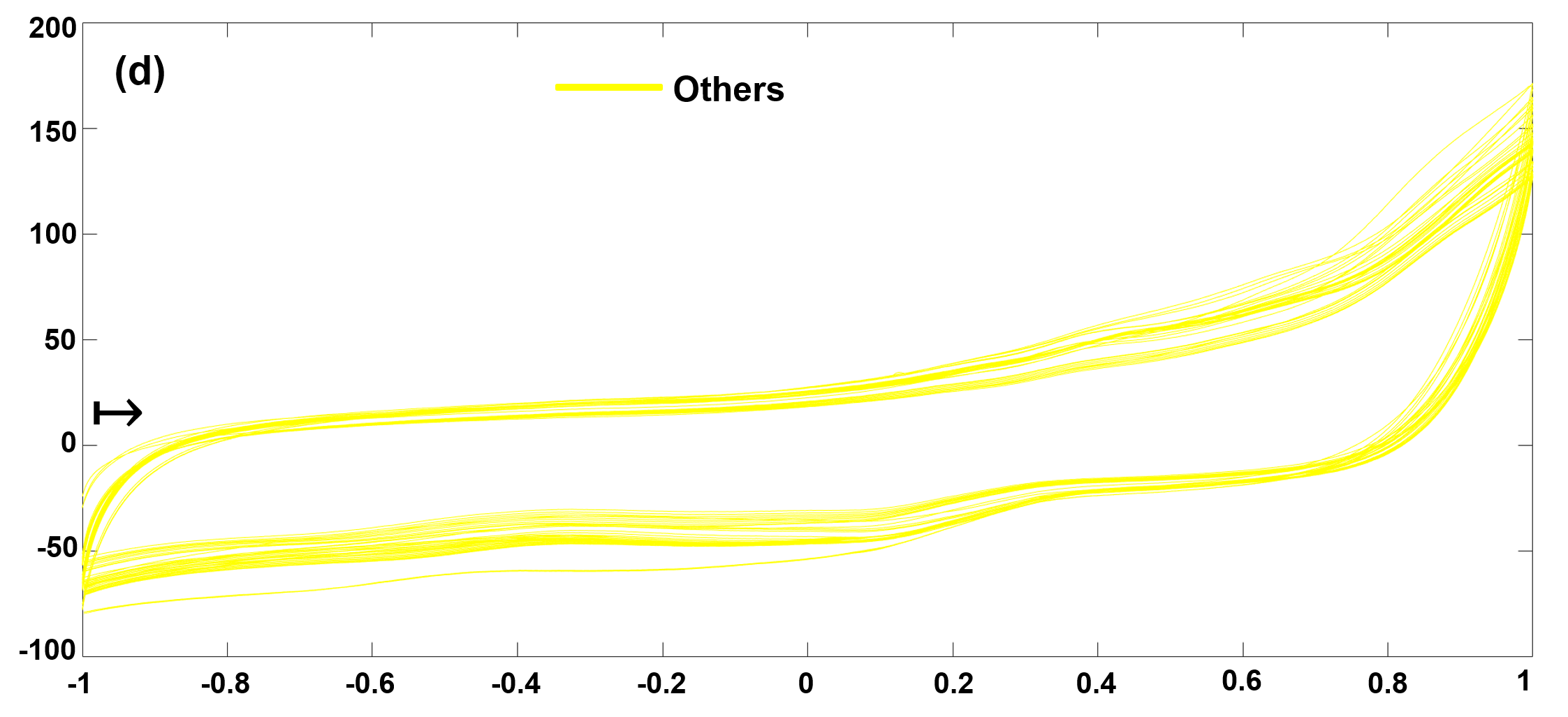


Fig. S4. Ciclyc voltamograms of different samples of beers from others obtained with SPCE. Scan rate 100 mV s^−1^ and potential windows of −1 to 1 V.

**Table S1 –** Number, brand, and manufacturers of the b*eer samples used in the study*

| **Commercial beer brand (coded)** | **Number of samples*** | **Manufacturer** |
| --- | --- | --- |
| **Sample A** | **15** | **D** |
| **Sample B** | **12** | **D** |
| **Sample C** | **10** | **A** |
| **Sample D** | **17** | **B** |
| **Sample E** | **12** | **C** |
| **Sample F** | **13** | **C** |
| **Sample G** | **8** | **B** |
| **Sample H** | **5** | **C** |
| **Sample I** | **7** | **B** |
| **Sample J** | **8** | **D** |
| **Sample K** | **15** | **A** |
| **Sample L** | **12** | **B** |
| **Sample M** | **16** | **A** |
| **Sample N** | **10** | **A** |
| **Sample O** | **17** | **B** |
| **Sample P** | **14** | **A** |
| **Sample X** | **34** | **A** |
| **Sample Y** | **28** | **C** |

# **Table S2** Performance parameters of the models against the training dataset for the classification models built for manufacturer and brand distinction

| **Technique** | **Manufacturer** | **Training** | | | |
| --- | --- | --- | --- | --- | --- |
|  |  | **Sensitivity** | **Specificity** | **Accuracy** | **IR** |
| **PLS-DA** | Manufacturer A | 0.94 | 0.96 | 0.91 | 0.17 |
|  | Manufacturer B | 1 | 0.96 |  |  |
|  | Manufacturer C | 0.74 | 1 |  |  |
|  | Manufacturer D | 0.91 | 0.95 |  |  |
|  |  |  |  |  |  |
| **SIMCA** | Manufacturer A | 0.97 | 0.96 | 0.93 | 0 |
|  | Manufacturer B | 0.91 | 0.99 | 0.97 | 0 |
|  | Manufacturer C | 0.88 | 0.93 | 0.92 | 0 |
|  | Manufacturer D | 0.88 | 0.97 | 0.85 | 0 |
|  |  |  |  |  |  |
| **SVM-DA** | Manufacturer A | 1 | 0.99 | 0.99 | 0 |
|  | Manufacturer B | 1 | 1 | 1 |  |
|  | Manufacturer C | 0.97 | 0.99 | 0.98 |  |
|  | Manufacturer D | 1 | 1 | 1 |  |
|  |  |  |  |  |  |
| **SVM-DA** | Brand X | 0.99 | 1 | 0.99 |  |
|  | Brand Y | 1 | 1 | 1 |  |
|  | Other brands | 0.99 | 1 | 0.99 |  |
|  |  |  |  |  |  |

**Table S3. Performance parameters calculated with the test dataset for the PLS-DA models built with a random permutation of the classes and authentic class model**

| Technique | Manufacturer |  |  | **Test** |  |  |
| --- | --- | --- | --- | --- | --- | --- |
|  |  | **Sensitivity** | **Specificity** | **Accuracy** | **IR** | **Q2y** |
| **PLS-DA** | Manufacturer A | 0.95 | 0.92 | 0.83 | 0.20 | **0.36** |
|  | Manufacturer B | 1.00 | 0.96 | 0.83 | - |  |
|  | Manufacturer C | 0.43 | 1.00 | 0.83 | - |  |
|  | Manufacturer D | 0.90 | 0.90 | 0.83 | - |  |
|  |  |  |  |  |  |  |
| **Permut 1** | Manufacturer A | **0.57** | **0.73** | **0.34** | **0.61** | **0.06** |
|  | Manufacturer B | **0.29** | **0.95** |  |  |  |
|  | Manufacturer C | **0.33** | **0.85** |  |  |  |
|  | Manufacturer D | **0.5** | **0.7** |  |  |  |
|  |  |  |  |  |  |  |
| **Permut 2** | Manufacturer A | **0.33** | **0.68** | **0.32** | **0.55** | **0.10** |
|  | Manufacturer B | **0.27** | **0.75** |  |  |  |
|  | Manufacturer C | **0.44** | **0.75** |  |  |  |
|  | Manufacturer D | **0.25** | **0.83** |  |  |  |
|  |  |  |  |  |  |  |
| **Permut 3** | Manufacturer A | **0.50** | **0.60** | **0.19** | **0.75** | **0.03** |
|  | Manufacturer B | **0** | **0.75** |  |  |  |
|  | Manufacturer C | **0.25** | **0.65** |  |  |  |
|  | Manufacturer D | **0** | **0.89** |  |  |  |

**
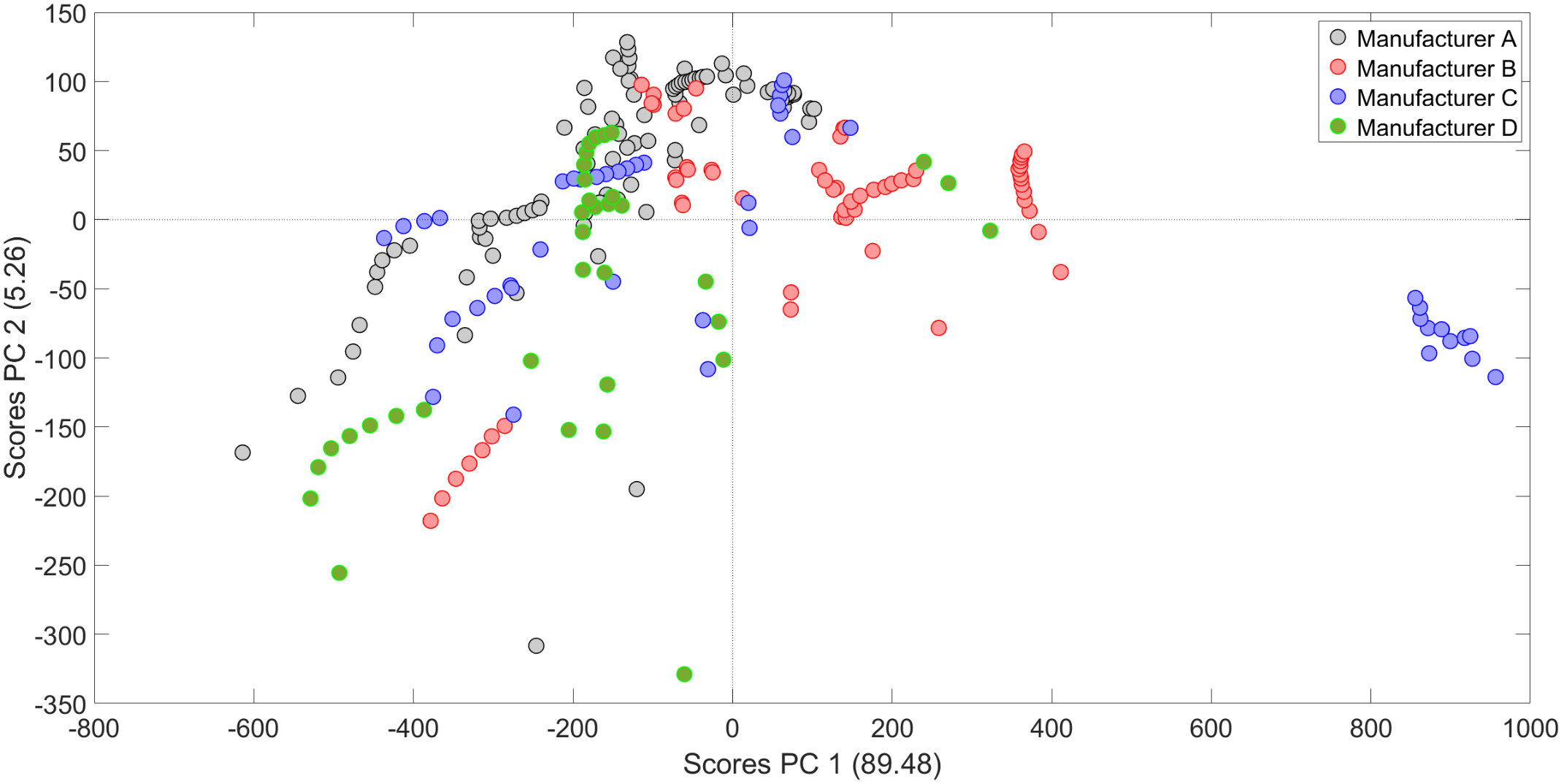
**

Figure S5 Principal component analysis scores plot from the carbon screen-printed electrode data for the four distinct Brazilian beer manufacturers
